# Transternal repair of a giant Morgagni hernia causing cardiac tamponade in a patient with coexisting severe aortic valve stenosis

**DOI:** 10.1186/1749-8090-6-30

**Published:** 2011-03-14

**Authors:** Ioannis Nenekidis, Vania Anagnostakou, Charalambos Zisis, Christos Prokakis, Efstratios N Koletsis, Efstratios Apostolakis, Panagiotis Dedeilias

**Affiliations:** 1 1st Cardiac Surgery Department, Evangelismos Hospital, Athens; 2 Radiology Department Evangelismos Hospital, Athens; 3 Thoracic Surgery Department Evangelismos Hospital, Athens; 4 Cardiothoracic Surgery Department, Patras University Hospital, Rio, Greece

## Abstract

**Background:**

Foramen of Morgagni hernias have traditionally been repaired by laparotomy, lapascopy or even thoracoscopy. However, the trans-sternal approach should be used when these rare hernias coexist with other cardiac surgical diseases.

**Case presentation:**

We present the case of a 74 year-old symptomatic male with severe aortic **valve **stenosis and global respiratory failure due to a giant Morgagni hernia causing additionally cardiac tamponade. The patient underwent simultaneous repair of the hernia defect and aortic valve replacement under cardiopulmonary bypass. The hernia was repaired through the sternotomy approach, without opening of its content and during cardiopulmonary reperfusion.

**Conclusions:**

Morgagni hernia can rarely accompany cardiac surgical pathologies. The trans-sternal approach for its management is as effective as other popular reconstructive procedures, **unless viscera strangulation and necrosis are suspected**. If severe compressive effects to the heart dominate the patient's clinical presentation correction during the cardiopulmonary reperfusion period is mandatory.

## Background

Morgagni hernias are very rare in adults accounting for 2-3% of all diaphragmatic hernias [1]. Although obstructive symptoms of the herniated viscera represent the most common clinical presentation there have been rare cases of severe compressive symptoms to the heart [2]. We present the case of a 75 year old male admitted to the hospital because of severe respiratory failure with cardiac tamponade due to a giant foramen of Morgagni hernia complicating an existing severe aortic valve stenosis. The patients underwent to emergency treatment of both problems under cardiopulmonary bypass. To the best of our knowledge this case is the only one reported with combined aortic valve replacement and Morgagni hernia repair.

## Case report

**Figure 1:**
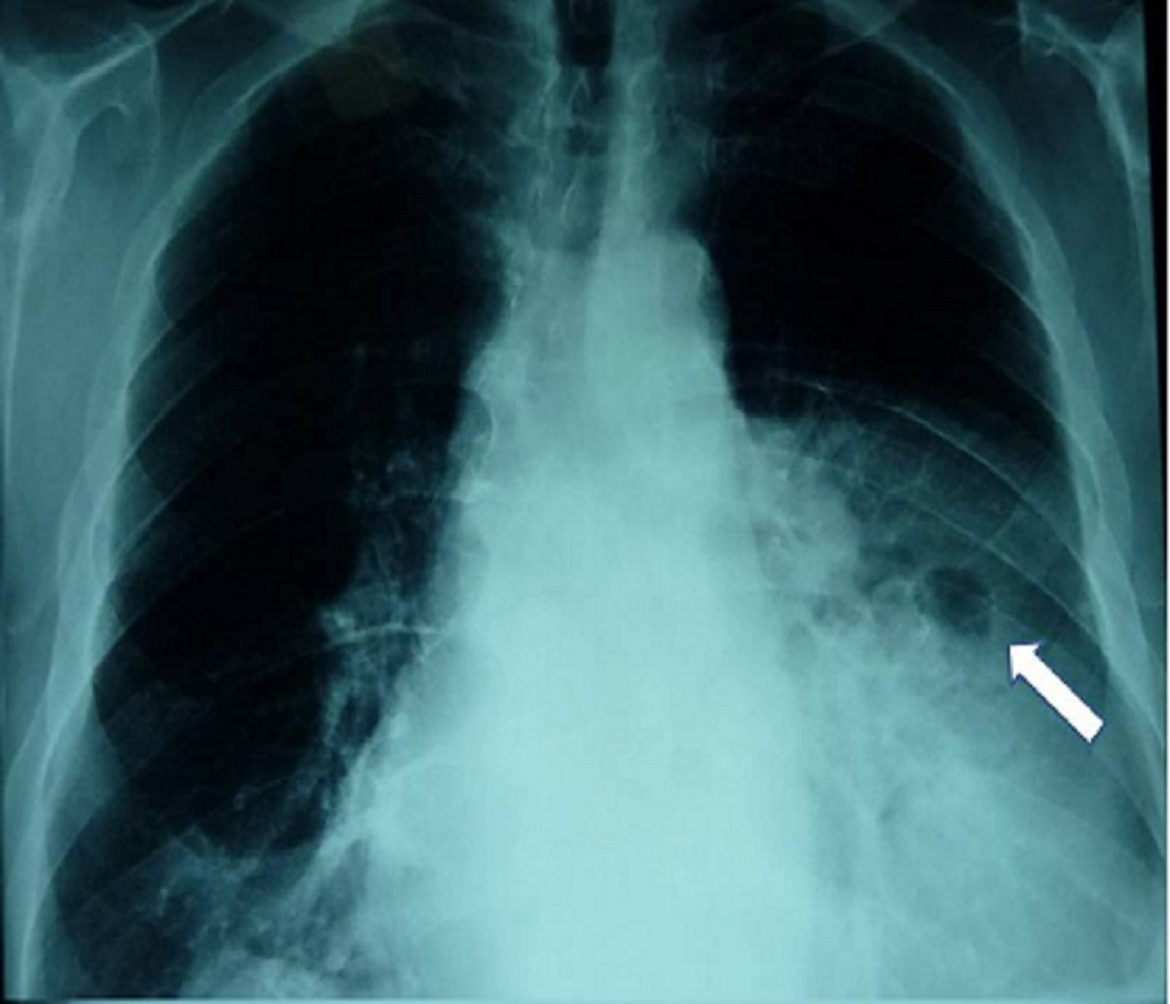
**Chest x-rays**. The arrow denotes the presence of air bubbles in the chest compatible with herniated viscera in the chest cavity.

**Figure 2:**
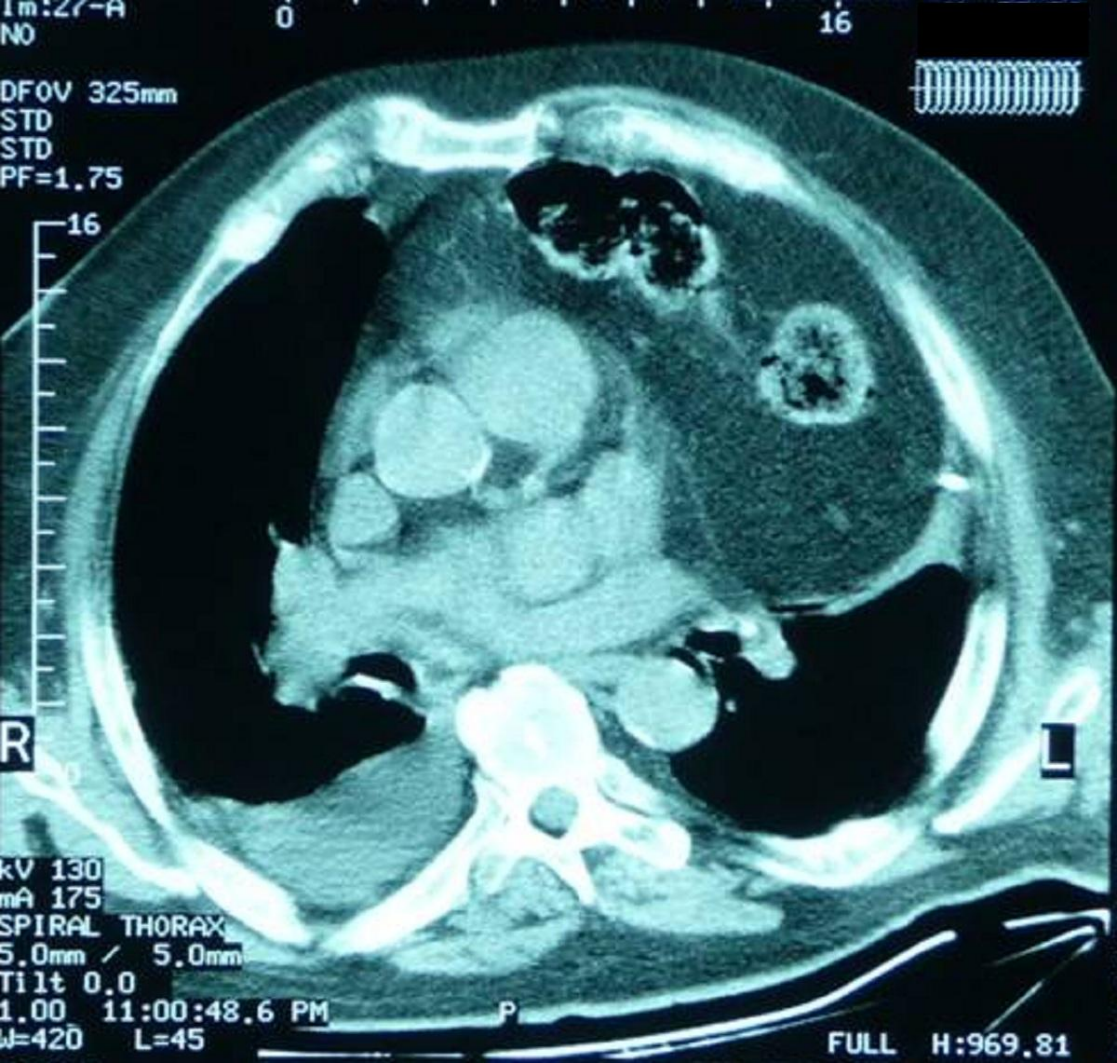
**Chest computed tomography imaging**. Both the omentum and the stomach protrude into the chest through the Morgagni's defect.

A 75-year-old obese man was admitted to the cardiac intensive care unit with fever (38.2°C), retrosternal pain and progressive dyspnea. The patient had distended jugular veins, paradoxical pulse wheezes and bowel sounds at the left hemithorax during auscultation. At the time of admission the electrocardiogram showed signs of left ventricular hypertrophy. **Chest **x-rays was remarkable for widening of the mediastinum compatible with the presence of viscera within the chest (Figure 1). Laboratory examinations included leukocytosis, increased CRP and INR of 1.5. The rest of his biochemical profile was normal and **full **blood count and coagulation profile were within normal limits. Blood gases indicated that the patient suffered from acute respiratory failure type 2 (PO2:65 mmHg, PCO2:51 mmHg, SatO2:89% under 100% oxygen supply) Echocardiography was hardly achieved due to presence of air within the anterior mediastinum. However a suspicion of cardiac tamponade was noted. Additionally severe aortic valvular stenosis due to significant valve calcification was revealed (Mean Gradient: 56 mmHg, Peak Gradient: 115 mmHg, **AVA 0.5 cm^2^**). Urgent computed tomography showed a giant Morgagni hernia provoking significant compression of the right ventricle. The hernia sac was adhered to the left lower lobe causing significant atelectasis. (Figure 2)

**Figure 3:**
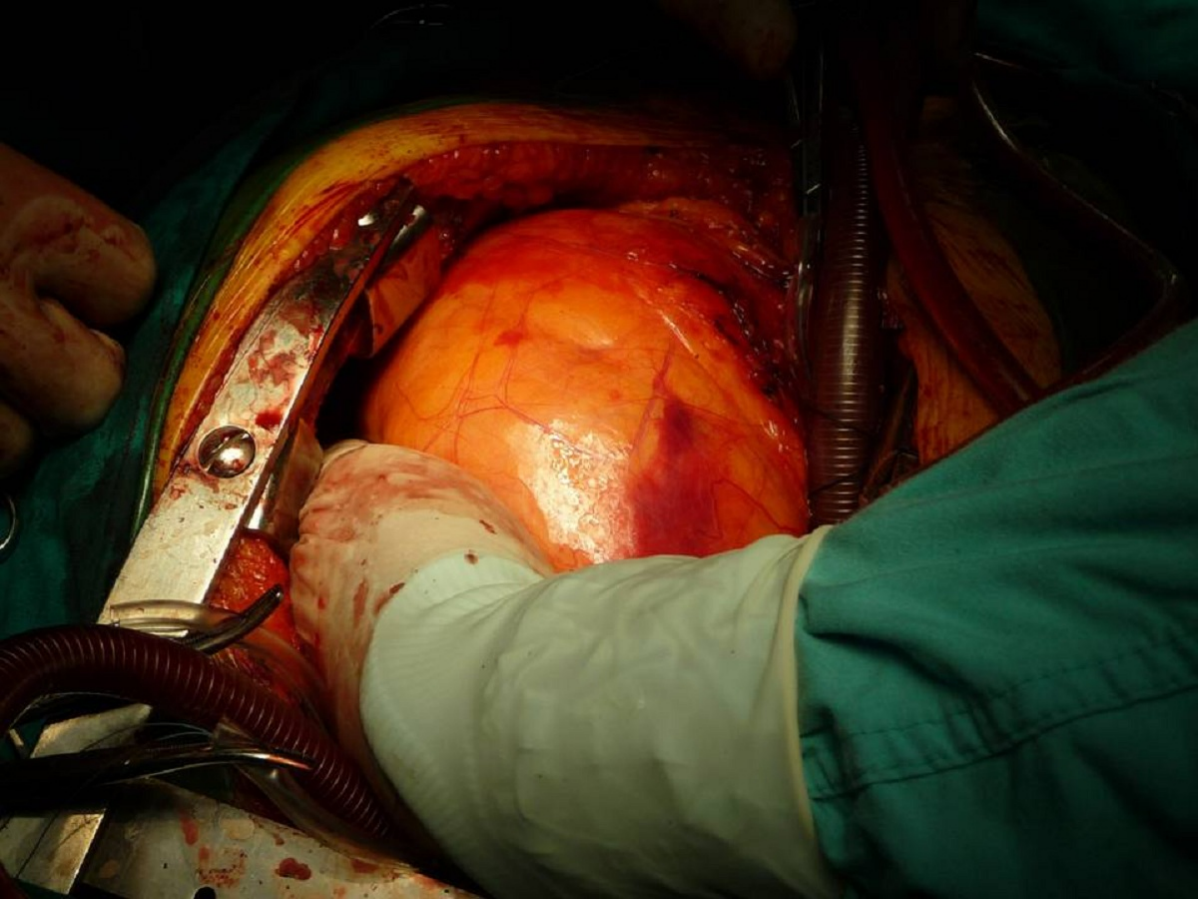
**The giant Morgagni hernia (intraoperative image)**.

**Figure 4:**
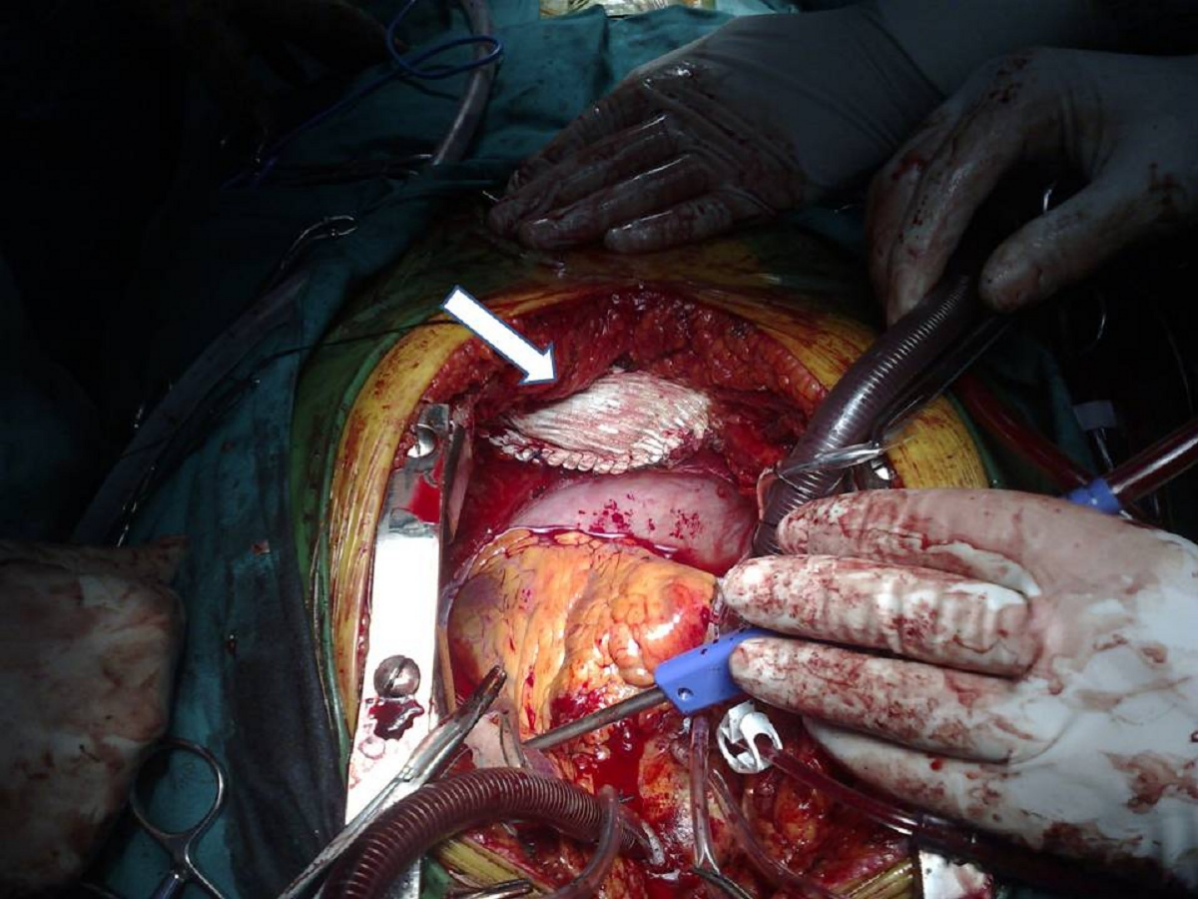
**Final reconstruction of the hernia**. A synthetic patch (arrow) was used to close the Morgagni's defect.

Two hours following his admission the patient was intubated and eventually underwent simultaneous surgical repair of the Morgagni hernia and replacement of the affected valve with a **bioprosthetic **one through median sternotomy. Initially aortic valve replacement was performed under cardiopulmonary bypass. **Lysis of the adhesions between the hernia sac and the lung parenchyma was necessary to relocate the protruded viscera into the abdomen without tendency **(Figure 3). Finally the distended foramen of Morgagni was reconstructed with a polypropylene patch which was sutured along the edges of the defected area. (Figure 4) The patient was extubated 10 hours later and he remained in the intensive care unit for 2 days. Bowel sounds became evident during the third postoperative day. Ten days after surgery he was discharged in good condition. Three months after discharge he remains free of symptoms.

## Discussion

Morgagni hernia is a rare malformation that constitutes 3% of all diaphragmatic hernias. It was first described by Giovanni Battista Morgagni in 1761. The foramen of Morgagni is a persistent developmental defect in the diaphragm anteriorly between septum transversum and the right and left costal origins of the diaphragm. A hernia through the foramen of Morgagni is invariably right sided and is presented as an anterior mediastinal mass. Though usually asymptomatic it may cause retrosternal pain, epigastric discomfort and dyspnoea. The content of the hernia is usually omental fat, while larger hernia may contain transverse colon, stomach or small intestine [3]. Echocardiography may show a right anterior pericardiophrenic mass. However in **this **case the **hernia sac was on the left side and the location of the stomach **in front of the heart made **very **difficult an accurate **echo **evaluation of the cardiac **function**. Further CT imaging diagnosed Morgagni's defect, defined its content as greater omentum and stomach and confirmed the severe compression of the right ventricle. In addition a severe aortic valvular stenosis complicated the diagnosis by worsening the clinical profile of the patient.

Up to now **there has been no report on a combined management of aortic valve stenosis and a Morgagni hernia. In this scenario the treatment should in generally be a two stage procedure**. The treatment of the severe aortic stenosis constitutes a priority towards any hernia defect **since **it threatens the patient's life and should be carried out immediately. In this case however the severity of the respiratory failure, due primarily to the compressive effects of the giant hernia, **dictated the need for an urgent combined management of both conditions**. The **cornerstone **of treatment was the rapid sternotomy and initiation of cardiopulmonary bypass so as to relief the obvious mechanical compression and cardiac tamponade provoked by the hernia.

Morgagni hernia is currently treated by laparoscopy, laparotomy or even thoracoscopy [4,5]. However the transternal repair of the hernia is preferred in patients undergoing concomitant open heart surgery [6-8]. The repair should be carried out during the cardiopulmonary reperfusion period in patients presenting such severe cardiac compression and every effort should be directed to secure hemostasis.

Conclusively, Morgagni hernia can rarely accompany several cardiac surgical pathologies. **Cardiac **surgeons should be familiar with the transsternal hernia repair which is as effective as other popular surgical reconstructive procedures, **unless gastric or bowel strangulation and necrosis are suspected**.

## Competing interests

The authors declare that they have no competing interests.

## Authors' contributions

All authors: 1) have made substantial contributions to conception and design, or acquisition of data, or analysis and interpretation of data; 2) have been involved in drafting the manuscript or revising it critically for important intellectual content; and 3) have given final approval of the version to be published.
